# Gut microbial influences on the adaptive immune system and the development of cow milk allergy

**DOI:** 10.5339/qmj.2022.fqac.17

**Published:** 2022-04-04

**Authors:** Tracy Augustine, Fariada Badri, Selvasankar Murugesan, Meritxell Espino Guarch, Mohammad Ameen Al-Aghbar, Rana El Nahas, Anthony Akobeng, Mamoun Elawad, Souhaila Al Khodor, Mehdi Adeli, Nicholas van Panhuys

**Affiliations:** ^1^Laboratory of Immunoregulation, Sidra Medicine, Doha, Qatar E-mail: nvanpanhuys@sidra.org; ^2^Microbiome and Host-Microbes Interactions Laboratory, Sidra Medicine, Doha, Qatar E-mail: nvanpanhuys@sidra.org; ^3^Division of Gastroenterology, Sidra Medicine, Doha, Qatar; ^4^Division of Allergy and Immunology, Sidra Medicine, Doha, Qatar madeli@sidra.org

**Keywords:** children, CMPA, gut microbiome

## Abstract

Allergic diseases constitute significant health and economic issues in both developed and developing nations, with epidemiological studies demonstrating a rapid increase in the global prevalence of food allergy among the pediatric population. Cow milk protein allergy (CMPA), one of the most common forms of food allergies observed in early childhood, affects between 2%–6% of infants and children under 3 years of age. CMPA can present as either an IgE-mediated atopic allergy or a non-IgE mediated allergic response. Antigen-specific T cells play a pivotal role in directing the type of inflammatory immune response that occurs as well as in the formation of immunological memory. IgE-mediated CMPA is thought to develop because of an abnormal expansion of allergen-specific type-2 helper T (Th2) cells and a corresponding deficiency in immune regulation by regulatory T cells (Tregs), thereby altering the Th2/Treg balance. The gut microbiota, established very early during childhood through host-microbe interactions, can influence the incidence of allergic diseases. In this study, we aimed to analyze both the microbiome composition and CD4+T cell differentiation patterns in pediatric patients with and without cow milk allergy to establish the association between these factors. Using 16S rRNA sequencing, we analyzed the microbiome composition in stool samples of allergic and non-allergic pediatric patients aged between 1–4 years and identified the microbial species abundant in IgE and non-IgE mediated cow milk allergies. To assess the CD4+T cell differentiation patterns, peripheral blood mononuclear cells (PBMCs) from these patients were re-stimulated with cow milk antigen, and T cell subsets were assessed using flow cytometry. Antigen-specific CD4+T cells were identified and sorted for high throughput sequencing and subsequent gene expression analysis. The CD4+T cell differentiation patterns of the total and antigen-specific T cells were analyzed and statistically compared with controls. The identification of the correlation between the CD4+T cell differentiation patterns and species-specific microbial abundance in IgE and non-IgE mediated cow milk allergies can help in determining how the gut microbiome influences the CD4+T cell immune compartment development, ultimately leading to the development of cow milk allergy in pediatric patients.

